# Recent Development of Flexible Tactile Sensors and Their Applications

**DOI:** 10.3390/s22010050

**Published:** 2021-12-22

**Authors:** Trong-Danh Nguyen, Jun Seop Lee

**Affiliations:** Department of Materials Science and Engineering, College of Engineering, Gachon University, Seongnam 13120, Korea; ntdanh041@gachon.ac.kr

**Keywords:** tactile sensor, flexible, piezoresistive, piezocapacitive, piezoelectric, triboelectric

## Abstract

With the rapid development of society in recent decades, the wearable sensor has attracted attention for motion-based health care and artificial applications. However, there are still many limitations to applying them in real life, particularly the inconvenience that comes from their large size and non-flexible systems. To solve these problems, flexible small-sized sensors that use body motion as a stimulus are studied to directly collect more accurate and diverse signals. In particular, tactile sensors are applied directly on the skin and provide input signals of motion change for the flexible reading device. This review provides information about different types of tactile sensors and their working mechanisms that are piezoresistive, piezocapacitive, piezoelectric, and triboelectric. Moreover, this review presents not only the applications of the tactile sensor in motion sensing and health care monitoring, but also their contributions in the field of artificial intelligence in recent years. Other applications, such as human behavior studies, are also suggested.

## 1. Introduction

Tactile sensation, one of the five senses defined by Aristotle, is caused by the excitement of a certain sensitivity in the skin [1,2]. Tactile sensing occurs when biological tissue movements, such as hair movement, or deformation or twisting of the mucous membrane of the skin, occur [3,4,5,6]. Specifically, when stimulation with mechanical energy is applied, the shape of the rear container located in the cell membrane changes, opening the ion passage. After that, depolarization occurs as the voltages inside and outside the cell change. This depolarization creates an activity potential, and the generated activity potential is transmitted as an electrical signal to the cerebrum along the axon of the nerve tax mark. With the development of technology, sensors that simulate the tactile transmission process have been developed due to the increase in demand for devices that can sensitively react to external stimuli [7,8]. The tactile sensor is a device that detects mechanical external stimuli (e.g., strain, pressure, humidity, sound, and temperature), and transmits an electrical signal [9,10]. The signal shows not only the relationship between the stimulus and the device, but also the properties of the stimulus. In detail, it provides data on the magnitude, shape, position, and distribution of forces derived from the tactile sense. Nowadays, scientists tend to develop their tactile sensors into multiarray devices. The structure of these devices usually contains many pixels, with the pixel size becoming smaller and smaller with the new technologies [11,12]. Therefore the sensor became more flexible with higher resolution. On the other side, sensitivity and the range of measurement also attached much attention [13].

To improve the performance of tactile sensors, many researchers have studied sensing materials acting as receptors [14,15,16,17,18]. For example, polarizable materials, such as barium titanate, have not only been made into nanostructures of various shapes, but have also been made into composite materials with other materials to improve the performance of sensing material. In addition, research efforts are being conducted to simplify the existing complex signal transmission system by changing the structure of the device, by varying the arrangement of transducers. In addition, tactile sensors that are capable of detecting various external stimuli at the same time by combining devices having different signal transducer systems are also being developed [19]. Depending on the material used to build tactile devices, there are various fabrication techniques. The molding method is the most well-known method, used to construct layers with a complicated structure such as a square block or pyramid [20,21]. Besides this, we also have a spinning method that was used to construct fiber structure including electrospinning or wet-spinning [22,23]. We also have some other known methods such as electrodeposition and chemical vapor deposition [24,25].

With the recent further development of technology, tactile sensors are being studied for their application in medical devices and artificial intelligence beyond conventional touch screens or motion sensing applications [26,27]. Tactile sensors required in the medical field are used in pulse oscillators, breast cancer screening devices, measurement of pressure distribution in humans, and pressure mats for preventing ascites [28,29,30]. In the case of artificial intelligence, tactile sensors with high spatial resolution and performance, such as the fingertips of robots, are required [31,32]. For the tactile sensor to be applied to the above application field, data must be obtained directly from the daily activities of the object being tested [33,34]. To this end, the tactile sensor device must overcome multiple sensor element arrangement and wiring processing problems, be implemented flexibly and thinly, and must also be attached to a wide free curved surface. In addition, since physical contact is made, it must be durable and be able to detect various physical quantities [35,36].

This review explains the different operating principles of tactile sensors, and presents the latest development trends to overcome the limitations of each principle-based device. In addition, specific applications in the fields of motion sensors, health care monitoring, and artificial intelligence, which are promising applications of tactile sensor devices, are introduced.

## 2. Working Mechanism of the Tactile Sensor

**Figure 1:**
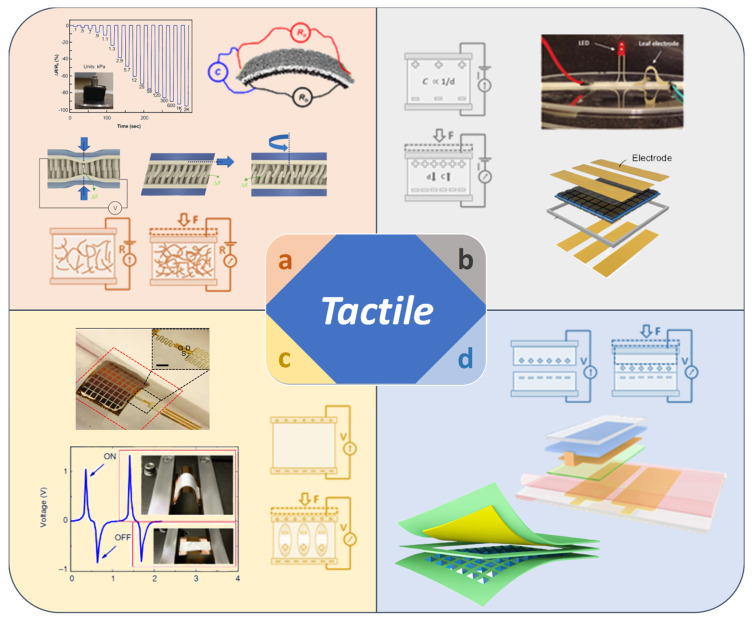
Working mechanism of different tactile sensors: (**a**) piezoresistive effect; (**b**) piezocapacitive effect; (**c**) piezoelectric effect; (**d**) triboelectric effect.

Tactile detection sensors have been developed to have an operating mechanism that is suitable for the structure and output signal of the device. The four types of operating principles currently used are piezoresistive, piezocapacitive, piezoelectric, and triboelectric. The first case exploits the piezoresistive effect that is strain-induced modulation of the conduction mechanism of a semi-conductor (Figure 1a) [37,38]. Transducers based on this effect usually have low impedance, high sensitivity, and a wide dynamic range. When the device undergoes deformation, the resistance is affected, therefore the output current also changes according to Ohm’s law. Transducers based on this effect usually have low impedance, high sensitivity, and a wide dynamic range. The change in ΔR in the electrical resistance R of the bar strained in the longitudinal direction is given by:

∆R = (1 + 2σ + M_i_) × R_χ_

where σ is Poisson’s ratio, χ is the longitudinal strain, and Mi is dimensionless longitudinal elastoresistance coefficient. The Mi is suggested as following:

M_ijkl_ = π_ijkl_ × Y

$$\pi_{\mathrm{ijkl}}=\Delta \rho /(\rho T)$$

and Y = T/χ

where Y is Young’s modulus, T is stress, and ρ is resistivity.

Secondly, the piezocapacitive effect is based on the working mechanism of a capacitor (Figure 1b) [39,40]. Specifically, when the physical structure of the dielectric affected by an external force changes, the output capacitance changes. The equation of working mechanism as following:

C = ε × A/d

∆C/C = ∆ε/ε_0_ + ∆A/A − ∆d/d_0_

with C is the sensing capacitance, A is the area of the capacitor, t is the thickness of the dielectric layer, and ε is the dielectric constant.

Thirdly, the piezoelectric effect is the internal generation reaction of an electric field according to a change in dipole moment resulting from a mechanical strain applied to a crystalline solid (Figure 1c) [41]. The most important factor in this effect is the change in polarization when an external force is applied, which can be caused by the reconstruction of the surrounding dipole, or the change of direction of the molecular moment [42]. Unlike the previous two cases, piezoelectric detection does not require a power supply to operate the device. The working mechanism is expressed as follows:

S = S_E_ × T + d × E

D = d × t + ε_T_ × E

where T is piezoelectric material’s stress, S is strain, D is charge-density displacement, E is the electric field, and d is matrix contains the piezoelectric coefficients for material [43].

Fourthly, the triboelectric effect is a type of contact electrification that occurs when a particular material is charged, after being separated from the surface of another material (Figure 1d) [44,45]. The basic operating mechanism is described as a charge in which a potential difference periodically occurs on the inner surfaces of two sheets with opposite directional triboelectric charges [46]. Charges are transferred by contact with these surfaces, leaving one as the anode, and the other as the cathode. When separated from the outside, an electric field is generated, and an output voltage is generated by the tactile device [47]. The working mechanism is as follows:

P_t_(t) = V_ab_(t) × I(t) = R × I^2^(t) + LI(t) × (dI(t)/dt)

where P is power output from the contact-separation mode triboelectric nanogenerator, R is the system inductance, and I denotes the current flowing the circuit [48].

### 2.1. The Piezoresistive Effect-Based Tactile Sensor

**Figure 2:**
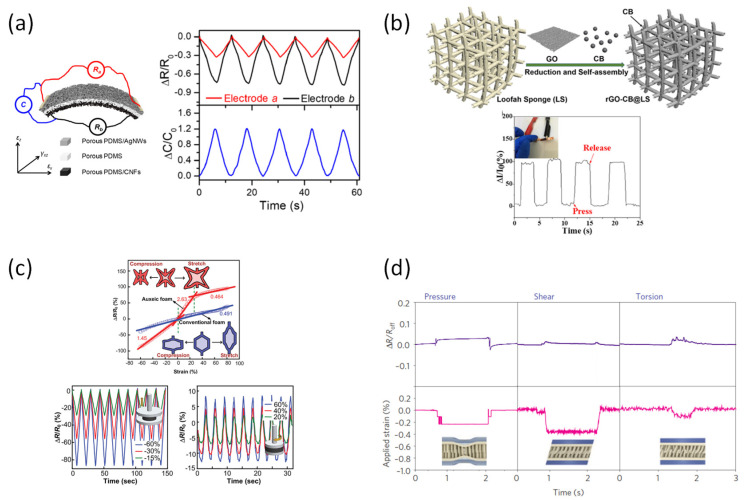
(**a**) Porous PDMS | PDMS/CNFs | PDMS/AgNWs piezoresistive sensor [50]. (**b**) Carbon black nanoparticles optimized 3D wearable graphene multifunctional piezoresistive sensor [51]. (**c**) Piezoresistive based CNT coated auxetic foams [52]. (**d**) Two interlocked arrays of high-aspect-ratio Pt-coated polymeric nanofibers supported on thin polydimethylsiloxane layer [53].

In recent years, piezoresistive devices have been developed based on various materials and device structures to detect changes of stimulus from the outside (Table 1). Previous studies have focused on generating signals according to changes in the energy band of semiconductors [49]. However, in recent years, studies of the application of new composite materials between conductive materials and insulating matrices have also been conducted. Peng et al. reported a device that included three stacks of porous polydimethylsiloxane (PDMS)/silver nanowire (AgNWs), PDMS/PDMS, and carbon nanofibers (CNFs) (Figure 2a) [50]. The sensor showed the ability to have high stretchable and capable of determining three forces simultaneously, namely normal pressure, lateral stretch, and transverse shear force. Due to the elasticity of PDMS and the conductivity via the conductivity of AgNWs and CNFs, it responded well to the changes in resistance while maintaining the strain.

With the development of 3D printing technology, it can be used today both with polymers and with composite materials to create very complex structures. Cao et al. used a 3D printer to develop an ultralight 3D hybrid piezoresistive sensor that was inspired by the loofah sponge and reduced graphene oxide (Figure 2b) [51]. Carbon black (CB) was introduced to optimize the conductivity of the reduced graphene oxide while enhancing the sensitivity of the multifunctional hybrid. The device is very small in size, but has high conductivity, short recovery time, excellent stability, and low manufacturing cost. Since traditional foam can provide excellent properties for piezoresistivity, the effect of the tunable Poisson ratio foam material on the piezoresistive effect was also investigated by Li et al. (Figure 2c) [52]. The negative Poisson’s ratio of the substrate significantly improves working performance in all three dimensions. Compared to the typical foam-based sensor, the auxiliary foam can both be extended laterally by an external force, and offer unique advantages to stretchable sensors and devices. These working conditions make the equipment more comfortable to accommodate the change of shape of a flexible area of the body.

To enhance the sensitivity of the piezoresistive effect, Pang et al. presented a layered strain-gauge sensor based on nanoscale mechanical interlocking between two arrays of metal-coated, high-aspect ratio nanofiber (Figure 2d) [53]. Mechanical sensing is based on numerous tiny contacts between the neighboring Pt-coated polymer nanofibers, which together created two layers of reversible electric interlocker. The multiplex, flexible strain-gauge sensor showed excellent response to pressure, shear, and even torsion, with a range smaller than 5%. Moreover, the output signals were proved to be able to maintain stability over 10,000 test cycles.

### 2.2. The Piezocapacitive Effect-Based Tactile Sensor 

**Figure 3:**
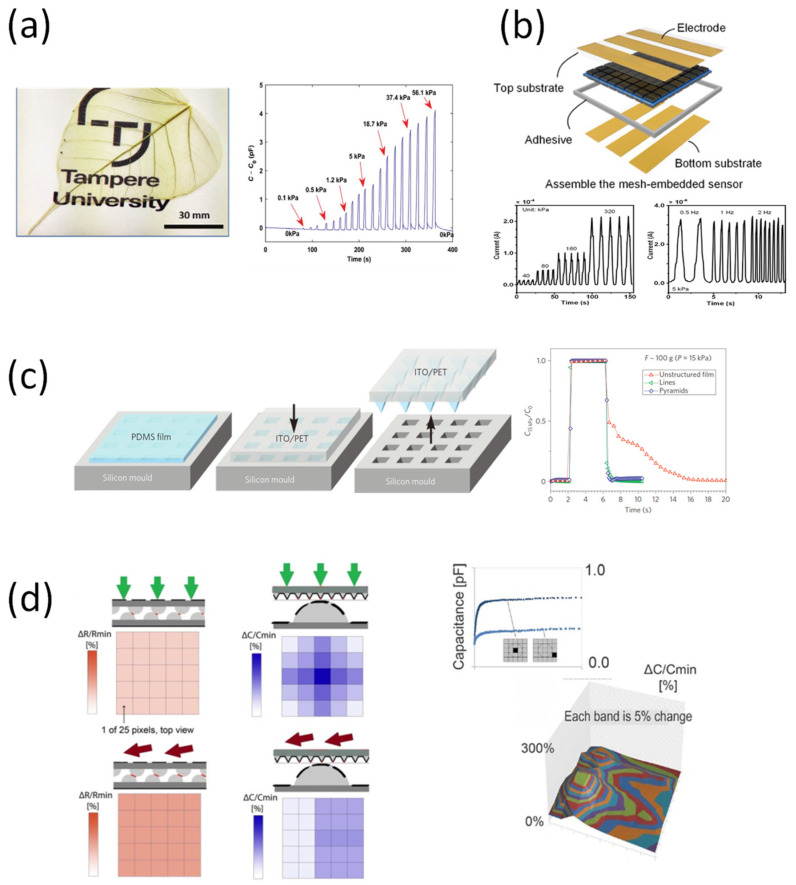
(**a**) Tactile sensor made from dielectric rose petal layer between two leaf electrodes [69]. (**b**) A proposed sensor utilizing fiberglass mesh [72]. (**c**) Pyramid microstructure PDMS films for piezocapacitance [73]. (**d**) Biomimetic e-skins with interlocked microstructure [76].

Due to the simple design, high sensitivity, and fast response characteristics of the capacitor, many studies have been conducted on the piezoelectric tactile sensor [68]. In addition, many methods have recently been proposed to improve wearability while improving the performance of the device (Table 2). Elsayes et al. introduced a plant-based device with the electrode built from leaf skeleton and rose petal as a dielectric layer (Figure 3a) [69]. Silver nanowires (AgNWs) were coated on the outside of the skeletal leaves to form a breathable and flexible interconnected conductive micro-network in the dielectric layer. On the other hand, rose petals that have undergone a freeze-drying process consist of a hierarchical microstructure 3D network, and serve as a compressible architecture that is responsible for sensing [70,71]. In addition, this composite material can be completely decomposed underwater during a short time of 75 days after use, so it can be applied as a disposable device. One of the other ways to increase the flexibility of the piezocapacitive sensor is to increase the number of pixels. Bae et al. introduced a flexible tactile sensor matrix pixelated using mesh layers (Figure 3b) [72]. The sensor matrix is uniformly patterned and electrically isolated between pixels using carbon nanotubes (CNTs) and polydimethylsiloxane (PDMS) composite materials. In addition, with the help of the mesh structure, confusion of the detection signal is prevented, and the detection layer is strengthened. Thus, this tactile sensor shows that the pressure exerted by the device is detected sensitively and independently.

Recently, other groups have attempted to combine piezocapacitive function with other elements, such as field-effect transistors (FETs). Mansfeld et al. proposed an organic thin-film pressure sensing device in which one of the core layers is a transistor structure, while the dielectric is made of rubber material (Figure 3c) [73]. The needle-like structure and flexibility of PDMS thin films allowed the device to measure human tissues and living cells [74,75].

The fast response time of the piezoelectricity sensor allows rapid collection of pressure information, and shows the possibility of application to artificial intelligence applications. Boutry et al. demonstrated a device that was capable of measuring and distinguishing both shear and pressing forces in real-time using a hierarchical pattern (Figure 3d) [76]. The structure of the sensor was created using the hierarchical structure of the sunflower to optimize the performance of the piezoelectric. Therefore, this sensor system not only detects small changes in the motion of the robot arm, but also suggests the possibility of controlling it for various purposes in the future [77].

**Table 2:** Comparative sensing performances of the piezocapacitive-based sensor.

Material	Structure	Application	Sensitivity	Advantage	Ref.
3D fabric	Planar fabric	Tactile system for robots	(0.86–2.50) fF kPa^−1^	Very sensitivity	[78]
Graphene	Planar	Pressor sensor array	6.55% kPa^−1^	Long time stability, position mapping, high resolution	[79]
AgNF-AgNW ^1^ and SiO_2_	Fiber	Pressor sensor array	1.78 × 10^−3^ ~9.65 × 10^−5^ kPa^−1^	Transparent, position mapping, high resolution	[80]
PDMS/Ecoflex ^2^	Planar/shoe sole	Plantar pressure sensor in shoes	6.8% N^−1^	Wearable, high sensibility, wide measure range	[81]
PDMS/Ecoflex	Planar/ pump bottom	Normal and shear force sensor	(2.5–3.0)% mN^−1^	High sensitivity, position mapping	[82]
Ag NWs/ PDMS/CPI ^3^	Lotus mold substrate/ AgNWs electrode	Tactile sensor for e-skin	1.2 kPa^−1^	Flexible, high sensitivity, high aspect ratio	[83]
Ionic liquid ^4^	Planar	3D force sensor	29.8 nF N^−1^	Be able to measure three-dimensional contact force	[84]
Ag/Ecoflex	Planar	Static and dynamic strain mapping	1.45 MPa^−1^	Wearable, dynamic strain mapping	[85]

^1^ AgNF-AgNW: Silver nanofiber-silver nanowire. ^2^ Ecoflex: Low viscosity, soft, platinum-catalyzed silicone rubber. ^3^ Ag NWs/PDMS/CPI: Silver nanowires/PDMS/Colorless polyimide. ^4^ Ionic liquid: 1-ethyl-3-methylimidazolium tricyanomethanide.

### 2.3. The Piezoelectric Effect-Based Tactile Sensor

**Figure 4:**
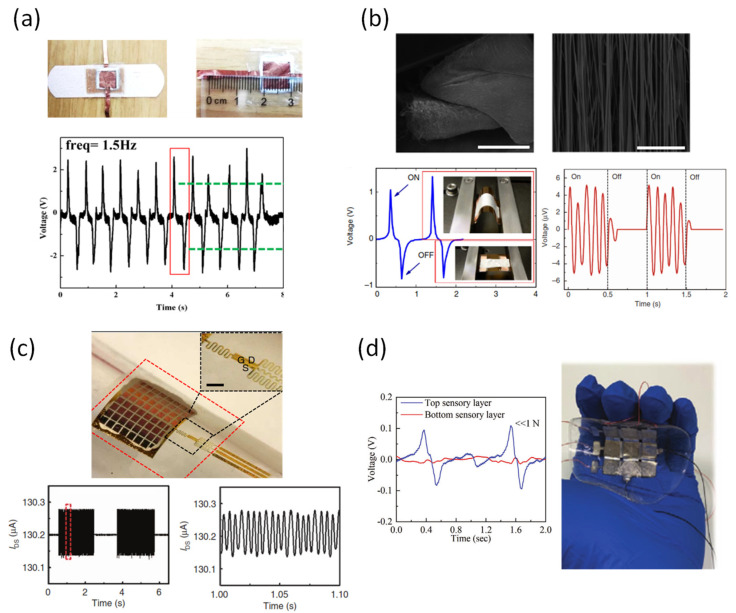
(**a**) Electrospun BaTiO_3_/Poly(vinylidene fluoride) nanocomposite membrane [89]. (**b**) Aligned arrays of nanofibers of poly(vinylidenefluoride-co-trifluoroethylene) for piezoelectric [90]. (**c**) Conformable amplified lead zirconate titanate (PZT) sensor [91]. (**d**) Crosstalk electrodes for spatiotemporally distinguishing diverse stimuli [92].

The piezoelectric effect is widely used in sensor, actuator, and transducer applications, due to the operating principle of generating electricity from external stimuli. Many studies have shown that dielectric materials are suitable for application to piezoelectric-based sensor devices (Table 3) [86,87]. As one of the dielectric materials, barium titanate (BaTiO_3_) is the most widely used substance, since it has environment-friendly properties, along with excellent dielectric constants and piezoelectric properties [88]. Furthermore, a composite material was produced using poly(vinylidene fluoride) (PVDF) to improve the physical properties of the BaTiO_3_. Jiang et al. proposed a piezoelectric sensor that was composed of PVDF fibers doped with BaTiO_3_ nanoparticles (BaTiO_3_NPs) using electrospinning (Figure 4a) [89]. In the composite fiber structure, BaTiO_3_ NPs also acted as nucleation sites for the formation of the β phase of PVDF, inducing the generation of high piezoelectricity in the device.

In addition, studies are being conducted to maximize piezoelectric performance by modifying the structure of polymer materials having dielectric properties. Chen et al. suggested the P(VDF-TrFe) (poly(vinylidene fluoride-co-trifluoroethylene)) aligned nanofiber-based tactile sensor using an electrospinning process (Figure 4b) [90]. The aligned polymer membrane-based tactile sensor showed an excellent piezoelectric effect (up to 40 nA and 1.5 V) with freestanding, high-density arrays. Moreover, this device presented a rapid response with periodical stimuli from any orientation.

To fully use the high conductivity and output power of the inorganic dielectric material, the development of a device through miniaturization of the inorganic element is in progress. To overcome the inflexible properties of inorganic ingredients, Dagdeviron et al. developed a design and theoretical model for ultra-small devices that can be attached to the skin (Figure 4c) [91]. This device consisted of a small-sized lead zirconate titanate (PZT) thin film on a soft substrate to provide stretchability. The compliant properties of the PZT thin film provide high performance without the interference of sensing signals at the same time as the rapid response of the equipment.

In addition to the development of flexible piezoelectric devices, studies of the arrangement of multi-pixel devices have been conducted. Lin et al. proposed a new piezoelectric flexible multifunctional tactile array that can distinguish various external stimulation modes in real-time using a simple but effective cross-talk free topology (Figure 4d) [92]. The designed tactile array has spatiotemporal detection and distinction ability of the magnitude, position, and diverse behavior, including touching, slipping, and bending.

### 2.4. The Triboelectric Effect-Based Tactile Sensor

**Figure 5:**
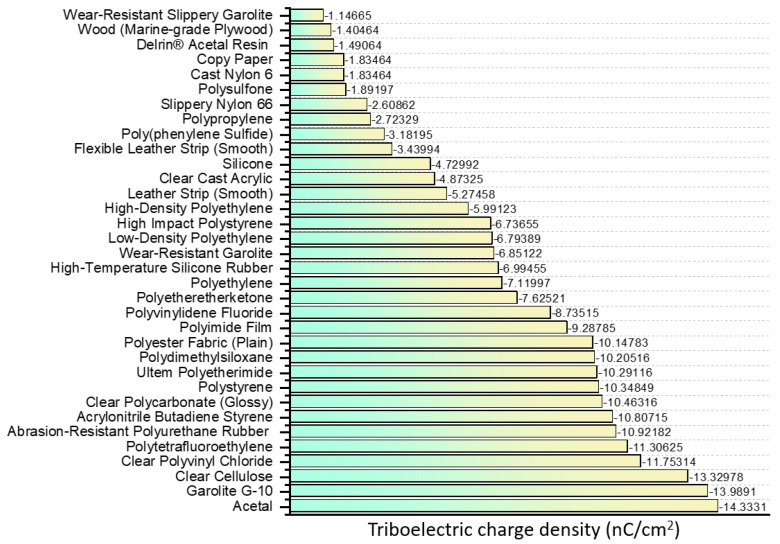
Familiar materials used for triboelectric and their triboelectric charge density [106].

**Figure 6:**
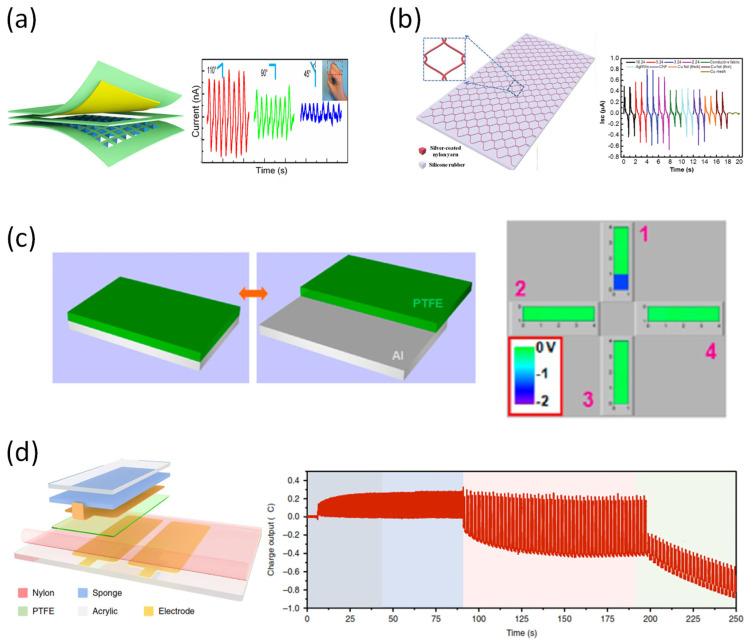
(**a**) Waterproof and stretchable triboelectric tactile sensor [108]. (**b**) Silver-coated nylon yarn on silicone rubber for skin-inspired triboelectric [109]. (**c**) Single-electrode based sliding triboelectric for vector sensor [110]. (**d**) Sliding mode triboelectric by charge space-accumulation effect [111].

The triboelectric effect was initially used as a power supply device, but due to its high efficiency, lightweight, low cost, environmental affinity, and usability at low frequencies, it is also used in tactile sensors (Table 4) [102,103,104,105]. To date, many studies have been conducted of the material showing a triboelectric effect and its charge density (Figure 5) [106]. However, not many studies have been conducted to apply the materials to the tactile sensor, due to its sensitive nature to environmental changes (e.g., presence of humidity, water, and dust on the friction surface) [107]. To overcome such problems, Chen et al. developed a fully encapsulated and scalable device (Figure 6a) [108]. The sensor device was made in the form of a single electrode consisting of polyurethane nanofibers (PUNFs) and silver nanofibers (AgNFs) covered with a PDMS container. This sensor device exhibits sensitive reactivity to friction acting from various angles, while preventing moisture penetration, due to the effect of the PDML layer.

In addition, sensor devices of various structures have been proposed to increase the elasticity of the electrode. Dong et al. developed a simple and flexible tactile sensor using a continuous chain link fence-shaped conductive silver-plated nylon yarn network (Figure 6b) [109]. The zigzag shape of the yarn plays an important role in improving the elasticity of the device. In addition, the location of the yarn is built between two layers of silicone rubber, therefore it has excellent sensitivity, high detection resolution, and fast response time. In addition, by constructing a pixel device, the fiber-based sensor can simultaneously shape the change in stimulation according to the location in 2D.

Since the two electrode systems of the triboelectric device cause signal interference due to the connection of the device, a device composed of one electrode is being developed to solve this problem. Yang et al. presented a single electrode-based triboelectric device based on periodic overlap and separation between a polytetrafluoroethylene (PTFE) and aluminum layers using relative sliding motion (Figure 6c) [110]. Specifically, a periodic change in the contact region between the polytetrafluoroethylene (PTFE) and an aluminum layer causes electrostatic and triboelectric effects, thereby generating movement of charges between the two layers. In addition, a single electrode-based device can be applied as a wireless sensor, due to its simple structure and operating principle.

Furthermore, research is being conducted to improve the output performance of the device by using operations such as electrode material change, surface modification, ion injection, and environmental control. He et al. expanded the charge movement space of the device by proposing a new structure of the sliding mode to improve the surface density of the electrode (Figure 6d) [111]. The device incorporates a structure between a shielding layer on a slider and an alternating blank-tribo-area to expand charge transfer. In addition, the structure can be expanded to be applied to a rotating device having high charging output density at low frequency.

### 2.5. Optical Tactile Sensor

Besides the electrical base tactile device, there is another stimulus detecting device type called an “optical tactile sensor”. The optical sensor uses materials that can generate light signals with different wavelengths, reflection, polarization, color, and intensity (Table 5) [123,124,125,126,127]. These devices have many advantages, such as low thermal noises and stray capacitances. However, they suffer from medium power required as well as integration complexity [128].

**Figure 7:**
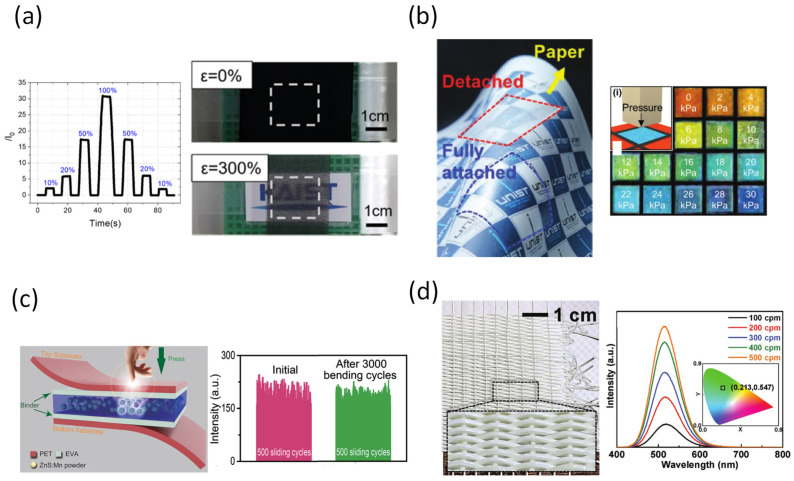
(**a**) Optical tactile device builds based on CNTs embedded in Ecoflex film [131]. (**b**) Adaptable and wearable photonic sensor based on shape-memory/responsive cellulose [132]. (**c**) Pressure mapping of personal handwriting device based on chemiluminescence [133]. (**d**) Self-power light-emitting fabric [134].

Gu et al. introduced a stretchable strain optical device based on the change of optical transmittance of the CNTs embedded on Ecoflex film (Figure 7a) [129]. Ecoflex material provides the sensor with outstanding mechanical properties including low Young’s modulus, good mechanical durability, and flexibility. Using spray-coating, multiwalled CNTs were applied on the surface of Ecoflex. The stretchability of the device can reach the point of 400% stretching with high sensitivity, stability, and reproductivity. The sensor was then demonstrated as a practical monitoring device applied to human skin. It is mentioned also that the sensor was able to distinguish the motion of finger-/wrist-bending, neck pulse/posture, swallowing, and face motion. Yi et al. presented an ultra-adaptable and stably wearable photonic skin (Figure 7b) [130]. The sensor can reversibly adhere to diverse substrates and visualize the external stimuli based on harnessing the liquid crystalline phase and amorphous phase of hydroxypropyl cellulose (HPC). The work also included an adhesive layer made from HPC, with the ability to change shape and shape recovery. The adhesive layer has been proven to have the capacity to be reused many times. Furthermore, since the HPC sensing layer reflects the light from the environment, the device does not require any additional power supply. The reflected light will change responding to the applied stress or strain of the HPC layer. However, the said advantage also means that the device cannot function when there is no light from the environment. The optional CNT electronic sensor in the middle of the device had confirmed that the optical sensing pad had high accuracy tower pressing force and strain.

A flexible pressure sensor matrix for recording both the handwritten graphics habits has been introduced by Wang et al. (Figure 7c) [131]. It is widely known that one of the most common methods for identifying verifications is an electronic signature according to the unique pressure/force applied on the sensing panel [132,133]. The device was built based on the ZnS:Mn particles as mechanoluminescent material sandwiched by polymeric layers. The applied pressure can provide energy for the particle so that it can emit the light with intensity responding to different strengths of the force, with a range from 0.6~50 MPa. Although the weak signal must be collected by a reading system, the response time is less than 10 ms with high spatial resolution. Jeong et al. demonstrated a mechanoluminescent (ML) fabric that can emit light using energy only from human motion (Figure 7d) [134]. In this case, the ML threads were woven into the textile, thus creating a wearable device that reduces energy waste. The binding of PDMS/ZnS with the fabricated cross shape fiber was improved by treating the fiber surface with primer solution. Finally, the fiber was coated with a silicon adhesive layer before being weaved into fabric. The fabric had proven to be a promising candidate for wearable displays with high-visibility outfits.

**Table 5:** Comparative of the recent optical-based sensor.

Material	Structure	Application	Sensitivity	Advantage	Ref.
poly(MAA-*co*-EDMA) ^1^	Monolithic Column	Detection and quantification of endogenous	N/A	No power source required	[135]
PDMS/ZnS:Mn	Multi array/ Pyramid	Pressor sensor	0.037~6 MPa^−1^	No power source required, high accuracy	[136]
CaZn_1–x_Mn_x_OS	Solid state	Color manipulation	N/A	High accuracy, good sensitivity	[137]
CaZnOS:Nd^3+^/epoxy	Composite disk	Force sensing	0~1000 N (Unknow minimum)	High accuracy, good sensitivity, fast respond time	[138]
Ca_2_Nb_2_O_x_:Pr^3+^ PVA ^2^	Pellets	Stress sensor	N/A	High accuracy	[139]
Ecoflex-ZnS PAM ^3^-LiCl	Planar	Pressor sensor	N/A	Stretchability, high accuracy	[140]
Electroluminescent	Fiber	Brain-interfaced communication	N/A	Stretchability, can show three color	[141]
ZnS PDMS	Planar	Stress sensor	N/A	Stretchable	[142]

^1^ Poly(MAA-co-EDMA): poly(methacrylic acid-co-ethylene dimethacrylate). ^2^ PVA: Polyvinyl Alcohol. ^3^ PAM: Polyacrylamide.

## 3. Applications

To date, studies on most sensor devices have been studied to provide more functionality, higher sensitivity, and better wearability. The most common application field for tactile sensors is motion sensors, and new devices, such as multi-pixel structures, are being developed to offer high sensitivity and wearability. In addition, the tactile devices can be applied to medical monitoring equipment and artificial intelligence devices using output signals from tactile sensor systems. Furthermore, recently developed tactile devices can distinguish external impact forces, and generate unique signals due to their high sensitivity. This improved performance increases the applicability of tactile sensor devices to other applications in the future, as they serve as both power and signal sources.

### 3.1. Motion Sensing-Robotic

**Figure 8:**
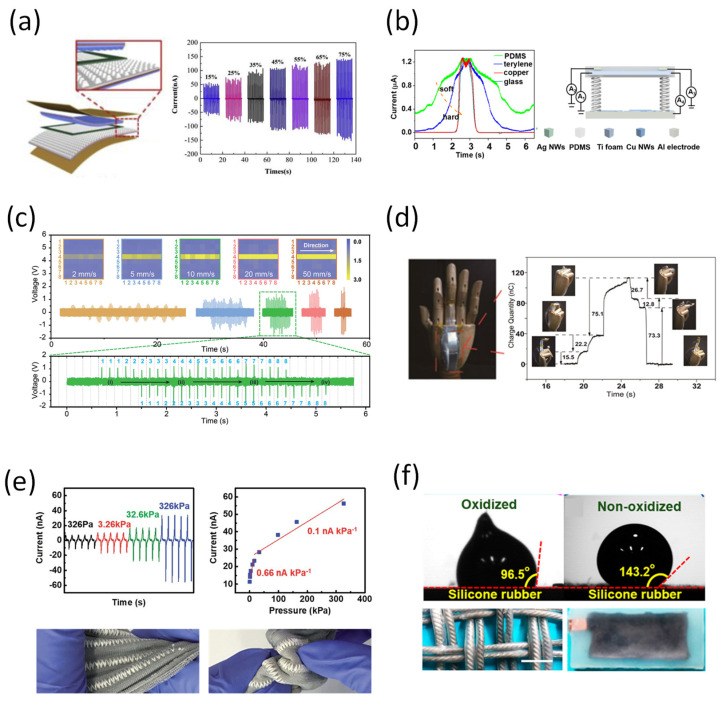
(**a**) Motion sensor constructs from Cu and P(VDF-TrFE) nanofiber pyramid electrode [143]. (**b**) Dual-mode triboelectric nanogenerator for location, pressure, and motion [144]. (**c**) Self-powered, high-resolution, and pressure-sensitive single electrode triboelectric generator based on single-electrode triboelectric generators that enable real-time tactile mapping [145]. (**d**) Triboelectric combined with artificial finger as a self-power sensing system [146]. (**e**) PVDF fiber thread sewed into a fabric using a sewing machine acted as TENG [148]. (**f**) Liquid-metal Galinstan electrode in silicone rubber triboelectric material for super stretchable TENG [149].

Detecting motion changes are the most representative application field of tactile sensors, and many studies are being conducted to achieve high sensitivity and a wide measurement range of devices. Recently, to introduce not only high sensitivity but also self-power, a device having two or more operating principles has been developed. As a specific example, a self-powered hybrid sensor that combines a piezoelectric and a triboelectric effect has been proposed to enable operation in a wide measurement range with high sensitivity. Yu et al. presented a hybrid sensor to detect physiological movement based on the contact–separation mode to combine piezo- and triboelectric effects (Figure 8a) [143]. With the external force applied to the device, the output signals are generated by the contact–separation process. The micro-frustum-arrays structure on the two friction layers consisted of P(VDF-TrFE), and lead zirconate titanate (PZT) membranes that have outstanding piezoelectric constants. The hybrid device showed stable piezo- and triboelectric effects under various frequencies, a large number of cycles, and humidity conditions. It also displayed good flexibility and conformal contact with irregular skin surfaces, as the elastic PDMS is used as the primary material. Li et al. proposed a smart self-power tactile device that can both detect a tiny applied pressure, and show the difference between the hardness of various contact materials (Figure 8b) [144]. By distinguishing the shape change of the current peaks, it is possible for the tactile device to create artificial skin that incorporates the feeling of the touched object. In detail, the single electrode unit acts as a contact sensor, while the two-electrode unit plays a key role in guiding the pressure. Thus, the structure of this device makes it capable of correctly identifying complex information from foreign impacts.

When the number of pixels is increased to make a high-resolution tactile sensor, the number of addressing lines increases, while the speed of signal processing decreases. To overcome this, Wang et al. have proposed a self-powered device with a new pattern for a high-resolution electrode based on a single electrode triboelectric generator (Figure 8c) [145]. These cross-type triboelectric sensor matrices can significantly reduce the number of addressing lines from (m × n) to (m + n) for a shorter measurement period for real-time mapping. Jin et al. reported a thermoelectric device that can emit different electrical signals depending on the angles of the foundation formed on the sensor (Figure 8d) [146]. Specifically, the charge and voltage generated by the equipment are independent of the frequency, and show different output values according to various movements of the finger. In addition, electrical signals generated according to movement in different joints appear differently. Therefore, using the principle of this device, it may be applied as a promising sensor in the robotics field of motion control.

The device based on fiber and fabric also seems to have promising potential for wearables sensors [147]. Shin et al. reported a mechanically strong PVDF fiber using “dry-jet wet spinning” method (Figure 8e) [148]. The authors mentioned the method to be similar to “wet-spinning” with the spinneret being outside the coagulation bath. The fabricated fiber’s properties are good enough to be used with a sewing machine. The stitching of PVDF threads enables has given advantages as flexible pattern design and form factors for motion sensing. It is also mentioned that the sensor has a very broad range of detecting capability (from 326 Pa to 326 kPa). Yang et al. developed a super-stretchable and structure-designable liquid-metal-based TENG (Figure 8f) [149]. The liquid metal Galinstan was injected into silicone rubber to form a flexible electrode. The rubber in the device structure plays the role of encapsulation and triboelectric material. This design has provided a large advantage in stretchability and functional up to 300% strain. Lastly, the sensor has been demonstrated to generate energy from human mechanical activity.

### 3.2. Health Care

**Figure 9:**
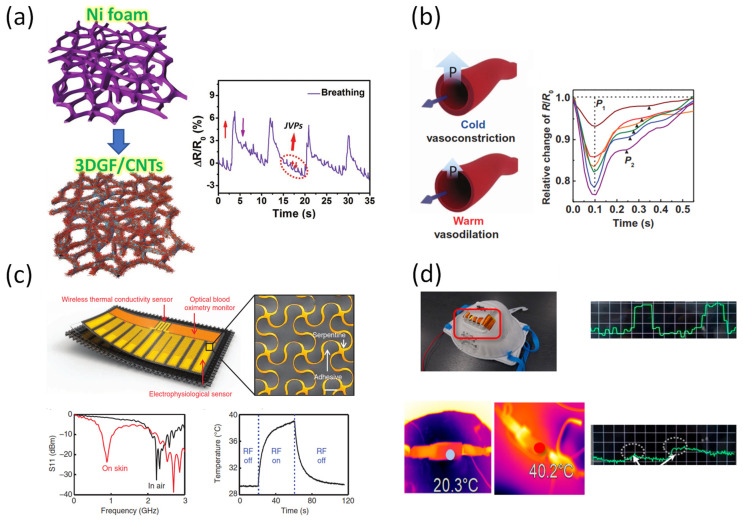
(**a**) All-carbon collaborative nanoarchitectures for epidermal sensors [150]. (**b**) E-skin tactile device for simultaneous monitoring of temperature and artery pulse pressure [151]. (**c**) Rugged and breathable electronics with an adherent composite substrate for transcutaneous monitoring [152]. (**d**) A triple-mode flexible e-skin sensor for wearable applications [153].

Tactile detection sensors are also applied to health care monitoring applications, including e-skin sensors, due to their high sensitivity. Since this field requires higher sensitivity than other applications, the piezoresistive and piezocapacitive effects should be used, rather than the piezoelectric and triboelectric principles of operation. However, the biggest weakness of devices using piezoresistive and piezocapacitive effects is that the signal-to-noise ratio (SNR) is generally low. To overcome this disadvantage, Cai et al. manufactured a 3D sensor substrate with carbon nanotubes attached to nickel foam using chemical vapor deposition (Figure 9a) [150]. The 3D micro-architectural carbon-based percolation network can provide sponge properties as well as electronic conductivity, which can detect large deformation. Moreover, the 3D carbon-based tactile foam sensor shows both high stretching properties and SNR, and high deformation resistance due to external stimulation.

Park et al. developed e-skin using graphene and PVDF composite by imitating the epidermis-skin microstructure of human skin (Figure 9b) [151]. When attached to the skin of the wrist, the interlocked e-skin may react to both temperature and arterial pulse pressure. In addition, the e-skin may recognize the time and space of static or dynamic tactile stimulation according to changes in arterial stiffness, pulse wave velocity (PWV), and reflected waves.

In e-skin applications that can measure health status in real-time, improving the flexibility of the device is an important factor for high adhesion. Jang et al. suggested a way to integrate elastic fabrics and thin electronic modules with flexible thermal and optical blood oxygen measurement and physiological monitoring systems (Figure 9c) [152]. Due to its high adhesion, this system both reacts sensitively to signals from the skin, even in hairy areas, and can be reused through detachment. In addition, the ultralow modulus silicone (UL–Sil) substrate used in the device showed good stability with respect to the electrode during stretching, while the finite element analysis showed little stress at the maximum strain of 60%. In addition, Kim et al. suggested a flexible reading integrated circuit that combines piezoresistive, piezoelectricity, and pyroelectric using graphene and PVDF as a substrate (Figure 9d) [153]. The flexible module operates by obtaining the desired signal by selecting an operation mode for the desired body part.

### 3.3. Artificial Intelligence

**Figure 10:**
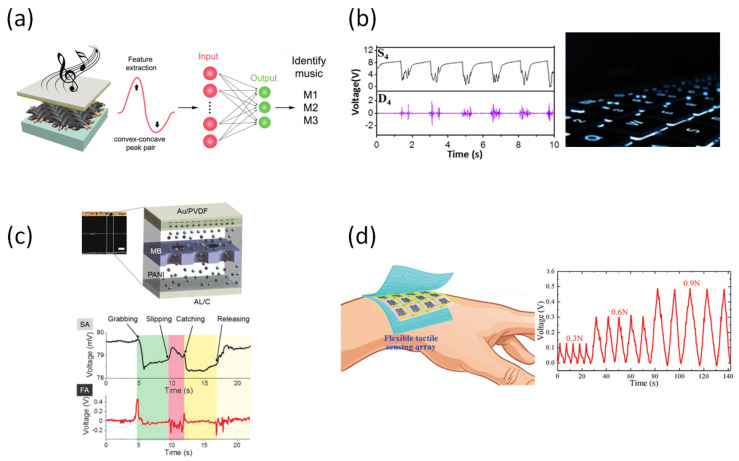
(**a**) A hybrid array on copper mesh sound sensor for artificial intelligence [154]. (**b**) Keystroke tactile for human-machine interface [155]. (**c**) Self-power sensor mimicking slow/fast-adapting cutaneous mechanoreceptors [156] (**d**) Highly flexible tactile sensor with an interlocked truncated sawtooth structure based stretchable rubber composites [157].

Recently, tactile sensors have been applied to artificial intelligence (AI) on external stimuli of artificial intelligence. Zhou et al. developed a flexible pressure sensor with a very short reaction and recovery time using the high surface area of the mesh substrate, and used it as a device to analyze the motion characteristics of the human hand (Figure 10a) [154]. Furthermore, it was also applied as a sensor capable of detecting the pressure of sound by using the principle that as the applied pressure increases, the current generated by the device increases. Data was collected based on a change in current according to the sound pressure collected by the device and then applied to AI training for song recognition.

Chen et al. proposed an intelligent keyboard device capable of identification according to a user’s touch (Figure 10b) [155]. The keyboard converts the typing operation into an electrical signal pattern, and then distinguishes the identity of the user according to the resistance change through a combined resistor. In addition, the electrical signal also records specific dynamic changes occurring during the input process for higher-level verification. Meanwhile, Chun et al. proved the correlation with substances in contact with human hands (Figure 10c) [156]. Specifically, by introducing an ion film into a piezoelectric device, the difference in reaction rate and the magnitude of the output signals were patterned using the principle of ion movement according to the injection of stimulation in the neuron system. As a result, the device formed a unique pattern, even in complex stimuli, such as gripping operation, sliding operation, and rubbing operation.

Furthermore, a tactile sensor having a biomimetic structure is being developed to collect response signals for various external stimuli. Wa et al. presented a highly flexible tactile sensor with an interlocked truncated sawtooth structure (Figure 10d) [157]. This structure allows the device to convert external pressure to internal tensile stress, which is the sensing mechanism of the graphene pattern. The graphene layer generates a small amount of voltage according to the conversion generated when the device system is pressurized. By using the signal change of the output voltage generated by this device, it is possible to distinguish the pattern of the touch applied to the device. 

**Figure 11:**
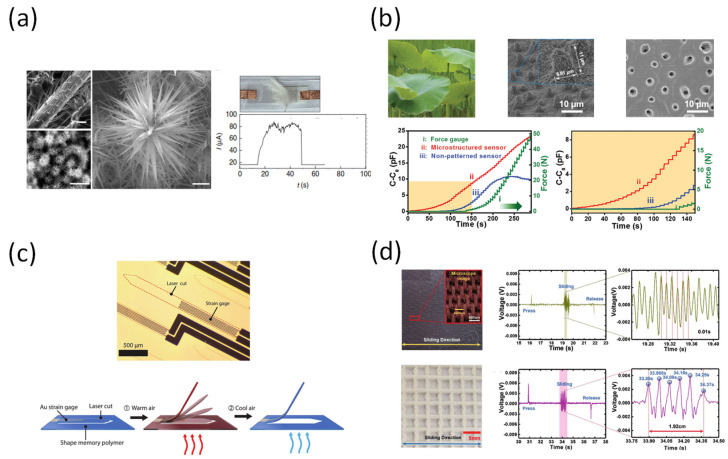
(**a**) A tactile sensor inspired by bristles or tactile hairs [159]. (**b**) Artificial intelligence based on the structure of the lotus leaf [162]. (**c**) Multimodal Electronic Whiskers [163]. (**d**) Tactile sensing system mimicking human tactile [164].

Some excellent structures in the natural environment can provide excellent response signals from the outside to the host, such as the ubiquitous tactile fur of the insect [158]. Li et al. mimicked the insect’s hair structure and function using spinal nanostructured zinc oxide fine particles (Figure 11a) [159]. Due to its unique structure that simulates insect fur, this device both offers fast response time and high sensitivity to external stimuli and provides excellent mechanical resilience [160,161]. Li et al. also produced a micro-patterned substrate similar to the structure of the lotus leaf and applied it as a tactile sensor (Figure 11b) [162]. Specifically, polystyrene microparticles were combined to the dielectric between the two electrodes of the device to form a sensor that exhibited high sensitivity to external stimuli, such as bending, stretching, and pressure. 

A scalable method for the 3D assembly of arrays that can be reconfigured post-fabrication has been demonstrated by Reeder et al. (Figure 11c) [163]. The adaptive electronic whiskers can sense a wide variety of stimuli using a single strain gauge. The e-whiskers are able to mimic the sensing capabilities of human feeling including proximity, applied force, and material texture, roughness. The sensor structure has allowed it to contain a very large number of pixels with high sensitivity. It has been proven that the device can even detect a human’s fingerprint. Kim et al. reported an artificial tactile sensor that can mimic the feeling of skin on different surface materials (Figure 11d) [164]. The human was sensed based on the piezoelectric multiarray system and the deep learning process. The authors chose a simple piezoelectric sensor to create the signal for the deep learning process. The systematic approach to mimic human tactile cognition was adopted, and thus make the sensor behave similarly to a human. From this study, we can see that tactile sensors can achieve remarkable results when combined with a good analysis system, thus becoming an outstanding candidate for development.

## 4. Conclusions and Outlook

In this review, we have investigated the tactile sensor and reviewed its development. Overall, the four major working mechanisms of piezoresistive, piezocapacitive, piezoelectric, and triboelectric were investigated. Their applications to touching sensors showed excellent working performance, and have thus furnished good results in motion sensing-robotic, health care monitoring, and artificial intelligence. In the future, advances in new materials, microstructure, and nanostructure can offer great potential for the improvement of sensors. On the other hand, artificial intelligence tactile device has got large achievements in advanced applications, such as behavior studies, or the identification of configurations. Yet, it still has so many promising potentials for further development. These developments can greatly speed up the growth of high technologies and human-machine interface studies.

## Figures and Tables

**Table 1:** Comparative sensing performances of the piezoresistive-based sensor.

Material	Structure	Application	Sensitivity	Advantage	Ref.
Metal alloy	Circular ultra-thin film	Tactile sensor	(0.76–4.18) uS/bar	High sensitivity	[54]
Cr/Au	Thick film cruciform Cantilever	Tactile force, shear stress, fluid flow	0.03 V/uN	Flexible High sensitivity	[55]
NiCr	Thin planar	Tactile sensor	Curvature: 40 ppm Force: 340 ppm/nN 230 ppm/µm	Stretchable	[56]
Zr_55_Cu_30_Ni_5_Al_10_ metallic glass	Flexible thin-film layer	Tactile sensor	N/A	Flexible High sensitivity	[57]
Graphene	Cylindrical film	Accelerometer	2.6 mV g^−1^	Ultralight High sensitivity	[58]
Carbon black silicone composite	Thick film	Pressure sensor	N/A	Stretchability High sensitivity	[59]
Polysilicon	Diaphragm	Pressure sensor	(3.35–3.74) mV/bar	Single-pixel mapping sensor	[60]
Silicone nanowire	Cantilever	Flow sensor	198 Ohm ms^−1^	High sensitivity wide measure range	[61]
rGO foam ^1^	Foam	Pressure sensor	22.8 kPa^−1^	Soft, high accuracy, sensitivity	[62]
GO-AgNF-PI ^2^ sponge	Foam	Pressure sensor	0.572 kPa^−1^	High sensitivity, accuracy	[63]
rGO/PU ^3^	Thin foam	Pressure sensor	0.67 kPa^−1^	Flexibility, high sensitivity, long time stability	[64]
Polysilicon	Silk surface	Monitoring human physiological signals	1.80 kPa^−1^	Wearability, high sensitivity	[65]
PEDOT:PSS ^4^	Nanofibers	Bending-insensitive pressure sensor array	N/A	Transparency, flexibility, wide measure range	[66]
Carbon	Planar electrodes, pyramid	Tactile sensor array	−1.10 kPa^−1^	Flexibility, position mapping, good accuracy	[67]

^1^ rGO: Reduced graphene oxide. ^2^ AgNF: Silver nanofiber. PI: Polyimide. ^3^ PU: Polyurethane. ^4^ PEDOT:PSS: poly(3,4-ethylene dioxythiophene) polystyrene sulfonate.

**Table 3:** Comparative sensing performances of the piezoelectric-based sensor.

Material	Structure	Application	Sensitivity	Advantage	Ref.
P(VDF-TrFE)/ BaTiO_3_ ^1^	Composite microfiber	Bending motion sensor for gesture	0.33 V kPa^−1^	Flexibility, high accuracy	[93]
BaTiO_3_	Fiber/fabric	Wearable physiological sensing	1.44 V N^−1^	Flexibility, High accuracy	[94]
P(VDF-TrFE)	Planar	Movement monitoring and e-skin	N/A	Transparency, high accuracy, fast response	[95]
Pb[Zr_x_,Ti_1−x_]O_3_	Thick block	Pulse monitoring	0.018 kPa^−1^	Ultrathin, high sensitivity, long time stability	[96]
PbI_2_	Planar	Motion sensor/ Environment vibration	N/A	Stretchability, long time stability, high sensitivity	[97]
PVDF ^2^	Nanofiber	Strain sensor	N/A	Stretchability, Wide measure range	[98]
PVDF	Thin film planar	Human motion	1.5 × 10^−5^ V mN^−1^	High sensitivity	[99]
PMMA ^3^ p-GaN	Vertical fiber Planar	Digital imaging	12.88 GPa^−1^	High resolution Flexibility	[100]
MoS_2_	Single atom layer	Energy conversion	N/A	Flexibility, Transparence,	[101]

^1^ P(VDF-TrFE): poly(vinylidene fluoride-co-trifluoroethylene). ^2^ PVDF: poly(vinylidene fluoride). ^3^ PMMA: Poly(methyl methacrylate).

**Table 4:** Comparative sensing performances of the triboelectric-based sensor.

Material	Structure	Application	Sensitivity	Advantage	Ref.
Hg/PVDF	Liquid metal/ Microfiber	Acceleration sensor	0.26 vs. m^−1^	Very high accuracy	[112]
PEDOT:PSS	Planar with wrinkle	Motion senor	(0.008–0.08) kPa^−1^	Stretchability, high accuracy	[113]
Silicone rubber/AgNWs	Nanowires sandwich by silicone	Motion sensor	0.034 V Pa^−1^	Stretchability, high accuracy, long time stability	[114]
PTFE ^1^/Cu	Gate for transistor	Touch tactile array system	(0.0778–1.028) mm^−1^	Flexibility, Long time stability, Position mapping	[115]
PVA ^2^	Microfiber on PDMS substrate	Touching sensor	4.4 Pa	Transparence, stretchability, long time stability	[116]
Graphene	Planar	TENG ^3^ mimics fast adapting of the neuron system	(0.04–1.64) kPa^−1^	High resolution, high sensitivity, position mapping, flexibility	[117]
ITO ^4^/PDMS	Planar/Pyramid on planar	Tactile sensor array	2.82 V Pa^−1^	Flexibility, wide measurement range, position mapping high accuracy	[118]
Fluorinated ethylene propylene	Planar with vertical fiber	Touching sensor	44 mV 1.1 V/Pa	Ultra-sensitivity, flexibility, transparency	[119]
PDMS PAAm ^5^–LiCl	Elastomers contain hydrogel	Flexible, transparent motion sensor	0.013 kPa^−1^	Ultra-stretchability	[120]
HCOENPs/BP/PET ^6^	PET fabric coat black phosphorus and particles	Motion sensor for artificial intelligence	N/A	Wearable, high accuracy, long time stability	[121]
PTFE	PTFE sandwich Cu	Water/air low sensor	N/A	High accuracy	[122]

^1^ PTFE: Polytetrafluoroethylene. ^2^ PVA: Polyvinyl alcohol. ^3^ TENG: Triboelectric nanogenerator. ^4^ ITO: Indium tin oxide. ^5^ PAAm: Polyacrylamide. ^6^ HCOENPs/BP/PET: Hydrophobic cellulose oleoyl ester nanoparticles/Black phosphorus/Polyethylene terephthalate.

## Data Availability

Not applicable.
